# Selective Labeling of Proteins on Living Cell Membranes Using Fluorescent Nanodiamond Probes

**DOI:** 10.3390/nano6040056

**Published:** 2016-03-25

**Authors:** Shingo Sotoma, Jun Iimura, Ryuji Igarashi, Koichiro M. Hirosawa, Hidenori Ohnishi, Shin Mizukami, Kazuya Kikuchi, Takahiro K. Fujiwara, Masahiro Shirakawa, Hidehito Tochio

**Affiliations:** 1 Department of Molecular Engineering, Graduate School of Engineering, Kyoto University, Nishikyo-ku, Kyoto 615-8510, Japan; sotoma.shingo.5x@kyoto-u.ac.jp (S.S.); jniimr@gmail.com (J.I.); igarashi.ryuji.78r@st.kyoto-u.ac.jp (R.I.); 2 PRESTO, Japan Science and Technology Agency, Kawaguchi 332-0012, Japan; 3 Institute for Integrated Cell-Material Sciences (WPI-iCeMS), Kyoto University, Kyoto 615-8501, Japan; hirosawa@frontier.kyoto-u.ac.jp (K.M.H.); tfujiwara@icems.kyoto-u.ac.jp (T.K.F.); 4 Department of Pediatrics, Graduate School of Medicine, Gifu University, Yanagido 1-1, Gifu 501-1194, Japan; ohnishih@gifu-u.ac.jp; 5 Graduate School of Engineering, Osaka University, Yamadaoka 2-1, Suita, Osaka 565-0871, Japan; smizukami@mls.eng.osaka-u.ac.jp (S.M.); kkikuchi@mls.eng.osaka-u.ac.jp (K.K.); 6 Immunology Frontier Research Center, Osaka University, Yamadaoka 3-1, Suita, Osaka 565-0871, Japan; 7 Department of Biophysics, Graduate School of Science, Kyoto University, Kitashirakawa-oiwake, Sakyo-ku, Kyoto 606-8502, Japan

**Keywords:** nanodiamond, nitrogen-vacancy center, polyglycerol, β-lactamase tag, membrane protein

## Abstract

The impeccable photostability of fluorescent nanodiamonds (FNDs) is an ideal property for use in fluorescence imaging of proteins in living cells. However, such an application requires highly specific labeling of the target proteins with FNDs. Furthermore, the surface of unmodified FNDs tends to adsorb biomolecules nonspecifically, which hinders the reliable targeting of proteins with FNDs. Here, we combined hyperbranched polyglycerol modification of FNDs with the β-lactamase-tag system to develop a strategy for selective imaging of the protein of interest in cells. The combination of these techniques enabled site-specific labeling of Interleukin-18 receptor alpha chain, a membrane receptor, with FNDs, which eventually enabled tracking of the diffusion trajectory of FND-labeled proteins on the membrane surface.

## 1. Introduction

Fluorescence imaging of proteins of interest requires highly specific molecular labeling with fluorescent probes, such as cyanine and rhodamine dyes, and fluorescent proteins [1,2,3], enabling visualization of the cellular localization of the proteins. Furthermore, through combination with single-molecule observation techniques, translational diffusion of receptor proteins can be tracked in the plasma membrane, allowing for characterization of their diffusion modes such as “hop diffusion” [4]. If two different fluorescent probes with different colors are conjugated to two different proteins, one can directly visualize their interactions in the cell [5], which is crucial for molecular understanding of various cellular phenomena such as guanine nucleotide-binding protein-coupled receptor activation [6] and the hand-over-hand walking model of the myosin V motor [7].

To observe the fluorescence from a single molecule, total internal reflection fluorescence (TIRF) microscopy has been developed [8] to monitor dynamics of biomolecules beyond the diffraction limit. However, the improvement of fluorescent probes does not meet the rapid advancement of these imaging techniques. For imaging, fluorescent dyes such as cyanine and rhodamine dyes are commonly used; however, these fluorescent probes show low photostability and thus result in bleaching of the intrinsic fluorescence [9,10]. This photobleaching property hinders long-term tracking of the probe. In recent years, semiconductor quantum dots (QDs) have been widely used in live cell fluorescence imaging and applied as single-molecule fluorescent probes, owing to their stability against photobleaching [11,12]. However, the strong photoblinking and cytotoxicity [13,14,15] associated with QDs limit their general applications, especially with respect to single-molecule tracking in living cells.

In this study, we examined fluorescent nanodiamonds (FNDs) as fluorescent probes for selective labeling of membrane proteins. FNDs fluoresce in the range of 600–800 nm [16,17], which arises from the nitrogen-vacancy centers (NVCs) existing as impurities in the diamond crystal structure. It is well known that the fluorescence from NVCs is highly stable and does not exhibit photobleaching or blinking [18,19]. Moreover, FNDs have been reported to display negligible cytotoxicity in various types of cells [20,21]. These properties render FNDs promising candidates for various types of cellular fluorescence imaging, including single-molecule tracking. In fact, an increasing number of reports utilizing FNDs have been published in the field of bioimaging in recent years [22,23,24]. However, their strategies for labeling biomolecules with FNDs mainly rely on antibodies against the target proteins [25,26,27]. Although antibodies can provide highly specific molecular recognition, their modification with FNDs is difficult to control as FNDs are conjugated to any lysine residues in the antibodies. Thus, it is difficult to create chemically uniform FND-labeled antibodies. The heterogeneity generated by the labeling method makes reproducible single-molecule tracking of proteins difficult. In addition, FNDs have unfavorable surface properties. Since the surface is generally covered by hydrophobic groups such as graphite [28], nonspecific adsorption of various biomolecules and self-aggregation can occur through hydrophobic interactions [25]. These features weaken the targeting specificity when labeling a specific single protein with an FND, distorting the obtained results. We previously reported that surface modification of nanodiamonds with hyperbranched polyglycerol (HPG) significantly suppresses the nonspecific adsorption of biomolecules as well as self-aggregation [25]. HPG modification-based selective targeting methods using the cyclic arginine-glycine-aspartic acid peptide and integrin receptor have been reported by Zhao *et al*. [29]. However, this targeting method does not form covalent bonds between the peptide and integrin and lacks versatility for bioimaging as it was not developed as a tag system. In this study, the HPG-modified FNDs are conjugated to proteins expressed on the plasma membrane via mutated β-lactamase-tag (BL-tag) [30,31]. BL-tag guarantees the generation of chemically uniform FND-labeled proteins on the membrane, while the HPG surface modification can provide an ideal dispersive nature without non-specific adsorption in aqueous environments. With this strategy, we successfully labeled the Interleukin-18 receptor alpha (IL18Rα) chain expressed on the plasma membrane of human embryonic kidney 293 (HEK293) cells. In addition, FND-labeled IL-18Rα was able to be tracked through single-molecule tracking techniques on the cells, revealing the excellent combination of FNDs with single-molecule techniques. The strategy opens up new avenues for cellular bioimaging of proteins.

## 2. Results and Discussion

**Figure 1:**
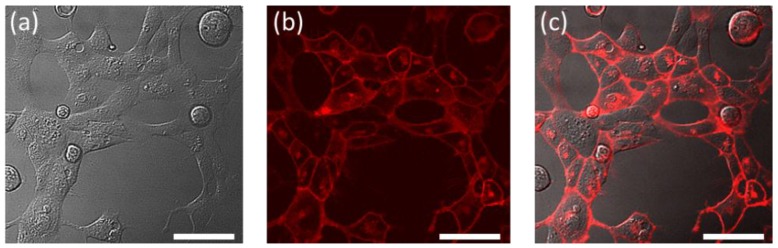
(**a**) Bright field image, (**b**) fluorescence image, and (**c**) merge of human embryonic kidney 293 (HEK293) cells after staining with ampicillin-modified tetramethylrhodamine (TMR). Scale bar shows 50 µm.

BL-tag is a mutant class A β-lactamase protein that was created to be covalently labeled with molecules with an ampicillin structure; *i.e.*, BL-tag binds the β-lactam ring in ampicillin, forming a covalent bond [30,31]. In order to achieve molecular specific labeling, we employed this system and applied it to IL18Rα, a membranous receptor protein for Interleukin-18, one of representative pro-inflammatory cytokines. First, we constructed a plasmid DNA encoding BL-tag-fused IL18Rα (BL-IL18Rα) and established an HEK293 cell line that stably expressed this protein on the plasma membrane. Stable expression of BL-IL18Rα in the cells was confirmed by fluorescence imaging after staining the cells with ampicillin-modified tetramethylrhodamine (TMR) [31]. This was accomplished by adding ampicillin-modified TMR to the culture media of the cells, followed by incubation for 30 min and subsequent washing with phosphate-buffered saline (PBS). The cells were then observed using a fluorescence microscope, which demonstrated that TMR-labeled IL18Rα were mostly localized on the plasma membrane (Figure 1).

**Scheme 1:**
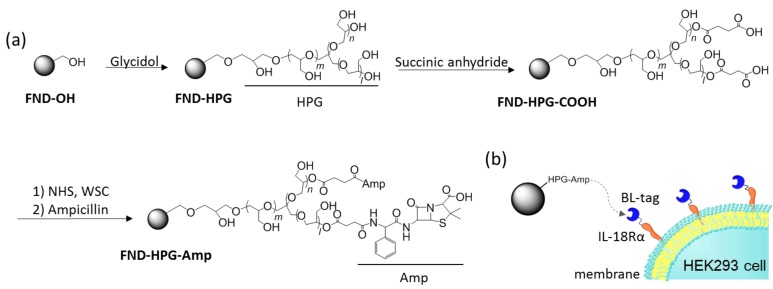
(**a**) Surface modification of fluorescent nanodiamonds (FNDs), FND-HPG: fluorescent nanodiamond-hyperbranched polyglycerol, HPG: hyperbranched polyglycerol, Amp: ampicillin, NHS: N-hydroxysuccinimide, WSC: water-soluble carbodiimide, FND-HPG-Amp: fluorescent nanodiamond-hyperbranched polyglycerol-ampicillin; (**b**) Scheme showing the specific labeling of Interleukin-18 receptor alpha (IL-18Rα) with FND using the BL-tag (β-lactamase-tag) system. HEK293 cell: human embryonic kidney 293 cell.

To label the BL-tagged proteins with 30 nm-sized FNDs, the FND surface was modified with HPG and the terminal COOH groups were further attached to the ampicillin moieties (Scheme 1a). The obtained HPG- and Amp-modified FNDs (FND-HPG-Amp) were expected to selectively react with BL-IL18Rα without nonspecific adsorption/aggregation (Scheme 1b).

**Figure 2:**
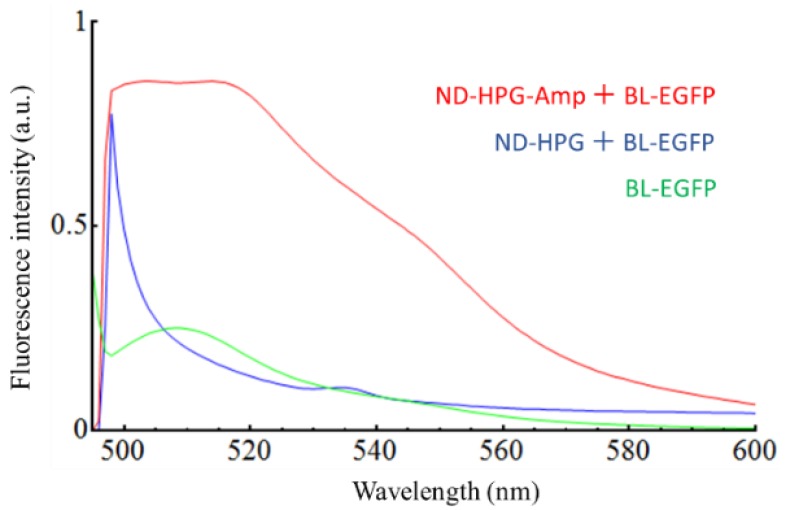
Fluorescence emission spectra of BL-tagged enhanced green fluorescent protein (BL-EGFP) (green), FND-HPG-Amp (red), and FND-HPG (blue) in Tris-HCl buffer after 2 h incubation with BL-EGFP and subsequent washing. Excitation wavelength was 488 nm. FND-HPG-Amp: fluorescent nanodiamond-hyperbranched polyglycerol-ampicillin; FND-HPG: fluorescent nanodiamond-hyperbranched polyglycerol; a.u.: arbitrary unit.

To verify the labeling selectivity of FND-HPG-Amp, we reacted it with BL-tagged enhanced green fluorescent protein (BL-EGFP) in a buffer solution in a test tube, in which 20 μL of the BL-EGFP solution (50 mg/mL) was added to 1 mL of Tris-HCl buffer (pH 8.0) containing 2 mg of FND-HPG-Amp. After 2 h of incubation at room temperature, the FNDs were collected by centrifugation and repeatedly washed until the fluorescence signal from the supernatant became negligibly low. The collected FNDs showed the characteristic fluorescence spectrum of EGFP (λ_ex_ = 488 nm, λ_em_ = 507–509 nm) [32], indicating that EGFP was successfully bound to FND-HPG-Amp via BL-tag (Figure 2, red). Note that the spectrum in the 500 to 525 nm region appears flat because of the detector saturation caused by strong fluorescence of the conjugated EGFP. Conversely, no fluorescence signal from EGFP was observed when FND-HPG was incubated with BL-EGFP (Figure 2, blue), indicating that nonspecific binding of BL-EGFP to FND-HPG was negligible. These results demonstrated that the BL-tag system with the ampicillin-modified FNDs was functional as intended. From the result, we roughly estimated that approximately two BL-EGFP molecules were conjugated to one FND-HPG-Amp particle (Supplementary Figure S1). In addition, we examined the feasibility of this FND-conjugation strategy *in situ*, in which the labeling specificity was demonstrated for a BL-tagged IL18Rα expressed in HEK293 cells (Supplementary Figure S2).

**Figure 3:**
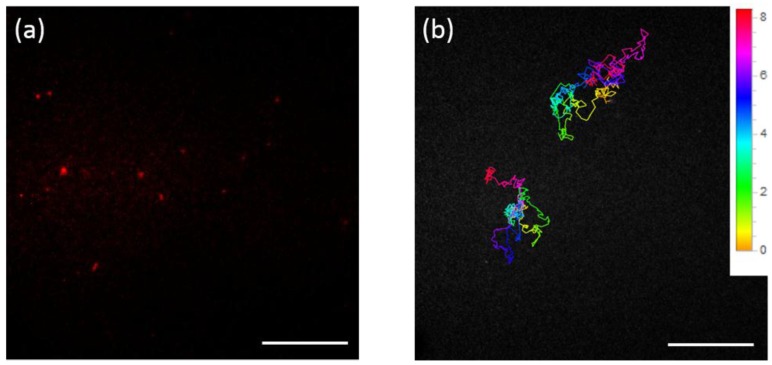
(**a**) Fluorescence image of the surface of a single cell treated with FND-HPG-Amp particles. Bright red spots indicate the fluorescence from FNDs; (**b**) Molecule trajectories of two FND-labeled IL-18Rα recorded for *ca*. 8 s. The color code of the trajectory shows the time course, as indicated on the right side. Scale bar shows 10 μm. Movies are provided in supplementary material.

We then tested whether the labeling strategy can be utilized in the fluorescence tracking experiments. The FND-HPG-Amp particles were reacted with BL-IL18Rα expressed on the membrane of living HEK293 cells. After extensive washing, fluorescence images of the cells were recorded immediately with oblique illumination fluorescence microscopy before the labeled receptors underwent internalization via endocytosis or cross-reacted with two or more proteins, as most of FNDs were expected to have more than one Amp moiety in our experimental condition. Figure 3a illustrates a typical fluorescence image of the surface of a single cell. Bright spots corresponding to FNDs were observed on the plasma membrane, which we tracked at a frame rate of 60/s. One may notice that the fluorescence intensity varied for each individual FND. This variation is due to the varied number of NVCs contained in each FND, but not indicating some FNDs aggregation giving rise to intense fluorescent spots. Namely, brighter FNDs contained more NVCs that emit fluorescence (Supplementary Figures S3 and S4). Figure 3b shows the molecule trajectories of FND-labeled BL-IL18Rα recorded for *ca.* 8 s (Supplementary Movie S1). Notably, during the 8 s tracking, the fluorescence emitted from the FNDs never bleached or blinked. In contrast, when Amp-conjugated TMR was used as a fluorescent probe for the single-molecule tracking of BL-IL18Rα, almost all fluorescent spots derived from the TMR-labeled BL-IL18Rα diminished in a few seconds, most likely because of bleaching (Supplementary Movie S2). From the trajectories of FND-labeled BL-IL18Rα, the mean squared displacement was calculated and the diffusion coefficient was determined to be 0.5 ± 0.1 μm^2^/s (*n* = 15). Note that the value is given as the mean ± standard error. This value is approximately consistent with that determined by using the TMR probe (0.4 ± 0.1 μm^2^/s, *n* = 9), and similar to those for transmembrane proteins laterally diffusing on the plasma membrane (0.09~0.59 μm^2^/s) [33].

A plausible application of our FND-labeling strategy would be in fluorescence imaging with one nanometer accuracy (FIONA) [7,34], which is a simple but powerful method to localize single fluorophores with nanometer accuracy in the *x*-*y* plane. However, with this technique, there is a trade-off between the spatial and temporal resolution. To achieve higher spatial resolution, acquisition of a large number of photons with longer exposure time is generally required, leading to lower temporal resolution. Thus, to attain both high spatial and temporal resolution, stronger laser irradiation is needed; however, this causes rapid photobleaching of the fluorophores. For instance, QDs bleach within 5 s under the 5 ms exposure times to obtain 1 nm scale accuracy [35]. In the case of FNDs, as the fluorescence does not bleach, stronger laser irradiation can be used; thus, higher spatiotemporal resolution can be achieved.

Another promising and attractive application of our strategy is in high sensitivity magnetic sensing in nanospace by employing the optically detected magnetic resonance (ODMR) of NVCs in FNDs. Electron spin resonance of an NVC can be optically measured under ambient conditions with high sensitivity at a single spin level [36]. Recently, it has been shown that ODMR can be widely utilized for magneto-, electro-, and thermo-sensing applications [37]. By combining our targeting strategy with ODMR techniques, nanoscale quantum sensing of such as conformational dynamics of a single protein in living cells can be achieved.

Although we obtained a reasonable value for the diffusion coefficient of the membrane protein in this study, it is still difficult to completely rule out the possibility that a single FND was connected with two or more proteins, as several ampicillin moieties could be loaded on the surface of a single FND in our preparation condition. To overcome this problem and create a FND-HPG particle labeled with a single ampicillin molecule, monofunctionalization on the nanoparticle surface can be used [38,39,40].

## 3. Experimental Section

### 3.1. Preparation and Surface Modification of FNDs

#### 3.1.1. Preparation of FNDs

In this experiment, 30-nm nanodiamonds (MD30, Tomei Diamond, Tokyo, Japan) were used. Ion irradiation, which is a common method for creating additional vacancies in nanodiamonds, was applied with He^+^ ions for 40 keV at a dosage of 1 × 10^13^ cm^−1^ [19]. The samples were thermally annealed at 800 °C under reduced pressure for 2 h in order to trap a moving vacancy with a substitutional nitrogen atom, leading to the formation of an NVC [17]. The samples were then oxidized in air at 550 °C for 2 h to remove the graphite on the nanodiamond surface [28]. To eliminate surface contamination, the samples were treated with a mixture of H_2_SO_4_:HNO_3_ (9:1 *v*/*v*) at 70 °C for 3 days and then centrifuged for 30 min at 15,000 *g*, discarding the supernatant. Next, 0.1 M NaOH was added and the suspension was stirred for 2 h at 90 °C. After centrifugation, the nanodiamonds were stirred in 0.1 M HCl for 2 h at 90 °C [41]. Finally, the nanodiamonds were centrifuged and washed three times with milli-Q water, yielding FNDs.

#### 3.1.2. FND-OH

FNDs were treated with borane in tetrahydrofuran (1 M) under nitrogen atmosphere with refluxing for 12 h. The reaction was quenched with an aqueous HCl solution and the samples were centrifuged and washed three times with milli-Q water, yielding FND-OH [42].

#### 3.1.3. FND-HPG

FND-OH was dispersed in glycidol (Sigma-Aldrich, St. Louis, MO, USA), sonicated for 2 h, and stirred for 10 h at 140 °C. The resulting gel was diluted with methanol and centrifuged at 87,000 *g* for 30 min to collect the hyperbranched polyglycerol-grafted FNDs (FND-HPG). FND-HPG was then centrifuged and washed four times to remove free polyglycerol [43].

#### 3.1.4. FND-HPG-COOH

FND-HPG was reacted with succinic anhydride (Wako Pure Chemical Industries, Osaka, Japan) in pyridine under argon atmosphere at 70 °C for 1 h. After the reaction, the samples were centrifuged at 87,000 *g* for 30 min to collect the COOH-terminated hyperbranched polyglycerol-grafted FNDs (FND-HPG-COOH). FND-HPG-COOH was then washed four times to remove excess succinic anhydride and pyridine [44].

#### 3.1.5. FND-HPG-Amp

FND-HPG-COOH (15.0 mg) was reacted with N-hydroxysuccinimide (NHS, 10.5 mg, Wako pure chemical industries) and water-soluble carbodiimide (WSC, 12.0 mg, DOJINDO, Kumamoto, Japan) in milli-Q water for 30 min at room temperature to activate the COOH groups. Ampicillin sodium salt (2.6 mg) was then added to the solution and stirred for 3 h. After the reaction, the samples were centrifuged at 87,000 *g* for 30 min to collect the ampicillin-modified FND-HPG-COOH (FND-HPG-Amp). FND-HPG-Amp was washed four times to remove unreacted materials. Conjugation of Amp to FND-HPG was checked by Fourier transform infrared spectroscopy (Supplementary Figure S5). After the surface modification, the hydrodynamic size of the particle enlarged to 80 nm (Supplementary Figure S3).

### 3.2. Cell Preparation

The DNA fragment of BL-tag fused between the signal peptide and the mature coding sequence of IL-18Rα (NM_003855, residues 1–19 and 20–542) was cloned into the pcDNA3.1+ plasmid vector (Invitrogen, South San Francisco, CA, USA). The plasmid vector was transfected to HEK293 cells (Japanese Collection of Research Bioresources, Osaka, Japan) using Nucleofector (Lonza, Basel, Switzerland). The cells were cultured in high glucose-containing Dulbecco’s modified Eagle’s medium (Invitrogen, Carlsbad, CA, USA) supplemented with 10% heat-inactivated fetal bovine serum (Sigma-Aldrich, St Louis, MO, USA), and geneticin (400 μg/mL). All cells were incubated at 37 °C in a humidified atmosphere of 5% CO_2_. The protein expression of BL-tag fused IL-18Rα for each colonized cells was confirmed with the western blot method.

### 3.3. Microscopy for Single Fluorescent Molecule Tracking of FND-Labeled Proteins

Single FND-labeled membrane proteins were visualized on the apical plasma membrane of HEK293 cells at 37 °C, using a homemade objective lens-type total internal reflection fluorescence (TIRF) microscope with an oblique-mode of illumination, based on a Nikon Ti-E inverted microscope (Tokyo, Japan) [1]. It was equipped with an oil immersion objective lens (Plan Apochromat 100× NA 1.49, Nikon, and a 580-nm dichroic mirror (FF580-Di01, Semrock, Rochester, NY, USA). The 561-nm solid-state laser (Jive, Cobolt, Solna, Sweden) was used to excite the FNDs and their fluorescence was detected through a long-pass filter (HQ650LP, Chroma Technology Corp., Bellows Falls, VT, USA). The fluorescence images were projected onto the two-stage microchannel plate intensifier (C8600-03, Hamamatsu Photonics, Shizuoka, Japan) coupled to a specially designed complementary metal oxide semiconductor (CMOS) sensor-based camera (Photron, Tokyo, Japan) by way of an optical fiber bundle, operating at 60 frames/s.

## 4. Conclusions

We established a method that allows for specific labeling of IL18Rα expressed on the plasma membrane with FNDs. HPG surface modification of FNDs dramatically decreased their nonspecific adsorptive nature, with specific conjugation between FNDs and IL18Rα being achieved via the BL-tag system. With this method, we successfully recorded the molecule trajectories of IL18Rα on the membrane. The labeling strategy described in this study can be widely applied to not only various membrane proteins such as receptors and ion channels but also cytosolic proteins. Considering the excellent fluorescence property of FNDs, high spatiotemporal resolution with extended recording time can be expected for various live cell fluorescence imaging techniques, including single-molecule tracking. Recently, NVC-based ODMR has been shown to provide unprecedented information for molecules in nanospace. Our labeling strategy allows for ODMR experiments on FNDs conjugated to specific proteins, which can offer unique information regarding the structure and dynamics of the proteins functioning in subcellular space, expanding new avenues for nanobiology.

## Supplementary Materials

The following are available online at http://www.mdpi.com/2079-4991/6/4/56/s1.
