# Supplementary material for: Selective Labeling of Proteins on Living Cell Membranes Using Fluorescent Nanodiamond Probes
Source: Nanomaterials (Basel). 2016 Mar 25;6(4):56. doi: 10.3390/nano6040056 (PMC5302567; doi:10.3390/nano6040056)
Supplement: Supplementary file 1 [file nanomaterials-06-00056-s001.zip › nanomaterials-113698--supplementary.done-final.pdf]

Supplementary Materials: Selective Labeling of Proteins on Living Cell Membranes Using Fluorescent Nanodiamond Probes

Shingo Sotoma, Jun Iimura, Ryuji Igarashi, Koichiro M. Hirosawa, Hidenori Ohnishi, Shin Mizukami, Kazuya Kikuchi, Takahiro K. Fujiwara, Masahiro Shirakawa and Hidehito Tochio

1. Number of BL-EGFP Molecules on One FND-HPG-Amp Particle

We tried to determine how many β-lactamase (BL)-tagged enhanced green fluorescent protein (BL-EGFP) molecules bound to one fluorescent nanodiamond-hyperbranched polyglycerol-ampicillin (FND-HPG-Amp) particle based on the fluorescence intensity of EGFP. Figure S1a shows fluorescence spectra of EGFP at various concentration in Tris-HCl buffer containing fluorescent nanodiamond-hyperbranched polyglycerol (FND-HPG) at a concentration of 2 mg/mL. Figure S1b shows the plot of the fluorescence intensity at 540 nm for each spectrum, which are fitted to a linear function. With assigning the value of fluorescence intensity at 560 nm for BL-EGFP bound to FND-HPG-Amp (Figure 2, red) to this line, we determined that roughly 9 μg of BL-EGFP were conjugated to 2 mg of FND-HPG-Amp in the experiment, yielding that a conjugating ratio of two BL-EGFP molecules on one FND-HPG-Amp particle. Note that the detector was saturated at the concentration above 10 μg/mL BL-EGFP in Figure S1.

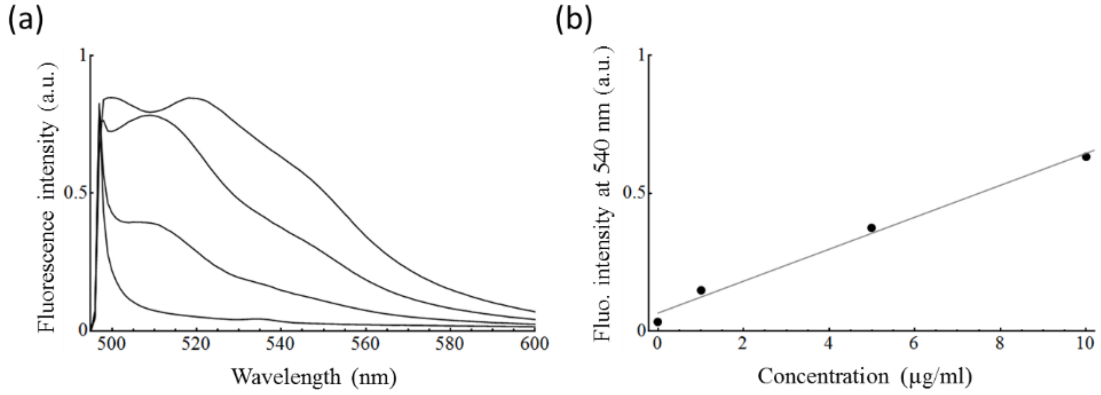

Figure S1. (**a**) Fluorescence spectra of enhanced green fluorescent protein (EGFP) at 0, 1, 5, 10 μg/mL in Tris-HCl buffer containing fluorescent nanodiamond-hyperbranched polyglycerol (FND-HPG) at a concentration of 2 mg/mL. Excitation wavelength was 488 nm; (**b**) The plot of the fluorescence intensity at 540 nm for each spectrum shown in (a). The line indicates the best fit to the data of a linear function. a.u.: arbitrary unit.

2. Specific Labeling of BL-Tagged Proteins with FND-HPG-Amp

We checked the specificity of our fluorescent nanodiamond (FND) labeling procedure as described below. Either FND-HPG-Amp or FND-HPG was added to human embryonic kidney 293 (HEK293) cells which were stably expressing β-lactamase-tag fused interleukin-18 receptor alpha (BL-IL18Rα) to a final concentration of 1 mg/mL. After the incubation for 30 min at 37 °C, the cells were washed with phosphate-buffered saline (PBS, pH 7.4) three times to remove the unbound FNDs, and the cells were imaged with a fluoresce microscope (Figure S2). Note that FND-HPG-Amp and FND-HPG were labeled with BL-EGFP and Fluorescein for visualization, respectively. Numbers of green fluorescent spots were observed on the cells treated with FND-HPG-Amp, whereas no or only very few such spots were found on the cells treated with FND-HPG. This result indicates that BL-IL18Rα was successfully labeled with FND-HPG-Amp. We also added FND-HPG-Amp to normal

HEK293 cells, however, only a few fluorescent spots were found, showing no non-specific adsorbent of FND-HPG-Amp onto the cell surface. These results demonstrate that our labeling strategy with the BL-tag system properly works *in situ* and can be used for cellular imaging.

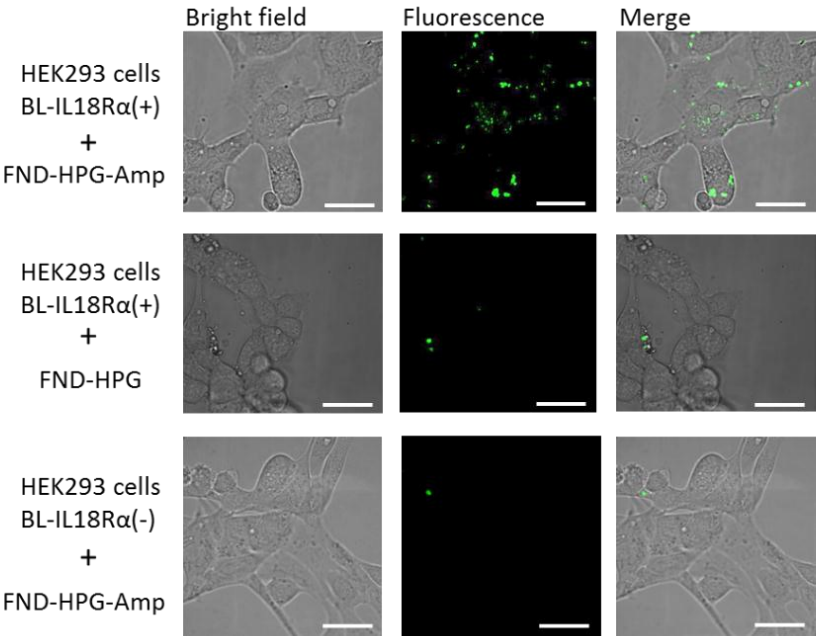

Figure S2. Bright field, fluorescence and merged images of HEK293 cells with or without stable BL-IL18Rα expression after a 30-min incubation with FND-HPG-Amp or FND-HPG and three-time PBS-wash. FND-HPG-Amp and FND-HPG were then labeled with BL-EGFP and Fluorescein visualization, respectively. Scale bar shows 50 μm.

3. Size and Aggregation States of FNDs

To confirm the size and aggregation state of FNDs before and after the surface modification, we measured dynamic light scattering (DLS). The size of as-received nanodiamonds were determined to be 37 ± 12 nm (median size ± standard deviation), whereas 52 ± 13 nm and 80 ± 21 nm for FND-HPG and FND-HPG-Amp, respectively (Figure S3). The coefficients of variance (CV), which is a good indicator to describe the aggregation state of dispersed nanoparticles in solution, were determined to be 32%, 25%, and 26%, respectively. Since the CV value did not increase after the surface modification, we believe that aggregation does not occur in our FND preparation.

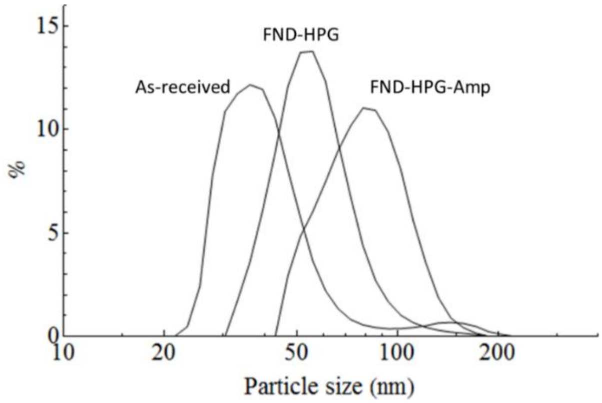

Figure S3. Size distribution of the FNDs with or without the surface modification determined by dynamic light scattering (DLS). As-received: as-received nanodiamonds without any surface modification.

4. Fluorescence Intensity of an FND

Figure S4a represents a typical fluorescence image of FNDs scattered on a glass plate, and the histogram in Figure S4b shows distribution of fluorescence intensity arising from FNDs. Although these FNDs were excited as an identical condition, emitted fluorescence intensity were significantly different for different particles. The difference is most likely attributed to variation in numbers of nitrogen-vacancy centers harbored in each FND.

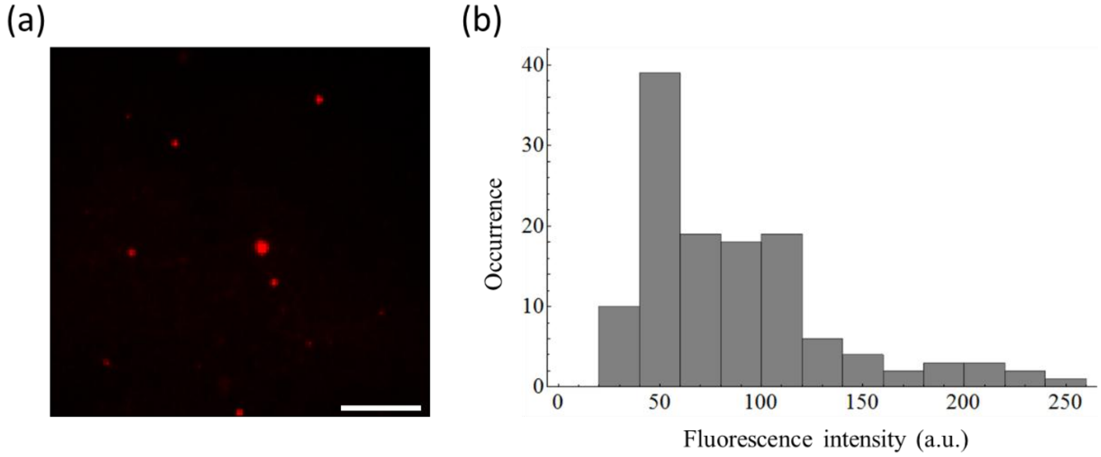

Figure S4. (**a**) Typical fluorescence image of FNDs scattered on a glass plate. Scale bar shows 10 µm; (**b**) Distribution of fluorescence intensity of FNDs.

5. Ampicillin Modification on Surface of FNDs

In order to confirm the ampicillin modification on the surface of FNDs, Fourier transform infrared (FT-IR) spectra of Ampicillin (Amp), FND-HPG-COOH, and FND-HPG-Amp were measured (Figure S5). Several Amp-derived peaks were observed in the spectrum of FND-HPG-Amp (e.g. 1601, 1416, 1297, 831 and 698 cm^{-1}), showing Amp was conjugated to FND-HPG. Based on the FT-IR spectra and fluorescence spectroscopy using BL-EGFP (Figure 2 in the main text), we concluded that the Amp modification was successfully accomplished.

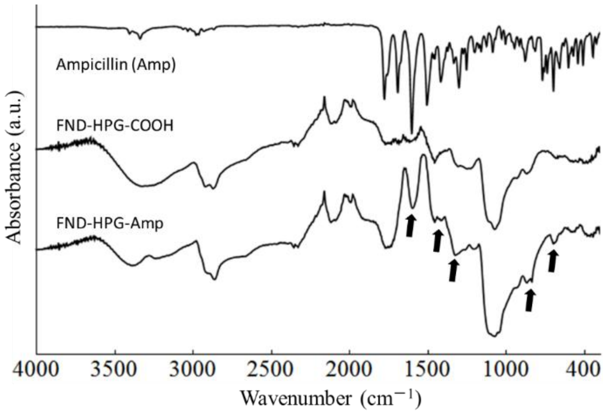

Figure S5. Fourier transform infrared spectra of the surface modified FNDs and ampicillin. Arrows indicate the typical Amp-derived peaks on FND-HPG-Amp.

© 2016 by the authors; licensee MDPI, Basel, Switzerland. This article is an open access article distributed under the terms and conditions of the Creative Commons by Attribution (CC-BY) license (http://creativecommons.org/licenses/by/4.0/).
